# LGI1 encephalitis manifesting as a delayed paraneoplastic response of squamous cell lung cancer on remission

**DOI:** 10.1093/omcr/omae171

**Published:** 2024-12-28

**Authors:** Ikechukwu Chukwuocha, Baig Al-Moyeed, Solomon Eigbe, Shilpi Shukla

**Affiliations:** Department of Neurology, New Cross Hospital, Royal Wolverhampton NHS Trust, Wolverhampton Road, Heath Town, West Midlands, WV10 0QP, United Kingdom; Department of Neurology, New Cross Hospital, Royal Wolverhampton NHS Trust, Wolverhampton Road, Heath Town, West Midlands, WV10 0QP, United Kingdom; Department of Neurology, New Cross Hospital, Royal Wolverhampton NHS Trust, Wolverhampton Road, Heath Town, West Midlands, WV10 0QP, United Kingdom; Department of Neurology, New Cross Hospital, Royal Wolverhampton NHS Trust, Wolverhampton Road, Heath Town, West Midlands, WV10 0QP, United Kingdom

**Keywords:** LGI1, autoimmune, behavioural changes, lung malignancy, FBDS

## Abstract

The leucine-rich glioma-inactivated protein 1 (LGI1) antibody-related autoimmune encephalitis can occur alone or in the setting of a malignancy and manifest with faciobrachial dystonic seizures (FBDS), cognitive decline, hyponatremia, and neuropsychiatric disorders. The importance of differentiating this entity from acute delirium cannot be overemphasized.

This review provides a detailed account of a 71-year-old man with previous diagnosis of lung cancer who presented with subacute onset behavioural changes, urinary retention, and FBDS. Investigation revealed hyponatremia, bilateral mesial temporal lobe high signal abnormality worse on the right on MRI and CSF positive anti-LGI1 antibodies (1:30). The patient was treated with immunosuppressive therapy with consequent symptom improvement.

This case emphasizes the need to have a high index of suspicion for this disease entity in patients presenting with new onset behavioural changes and the importance of identifying the typical FBDS, as early initiation of treatment confers a positive outcome for diseased patients.

## Introduction

Several neural-specific autoantigens have been linked to autoimmune limbic encephalitis (LE); encephalitis related to anti-LGI-1 antibody was first reported in 2010 [1]. with majority of patients exhibiting LE, clinically characterised by a subacute disruption in behaviour and memory that frequently occurs in conjunction with seizures. Immunotherapy tends to help patients, but its long-term results are not well described, especially in patients with malignancy [2].

Anti-LGI1 encephalitis is highly specific for FBDS, although it is only found in a small percentage of patients. These are 1–2 seconds involuntary contractions that affect the unilateral arm, leg, and face and can occur up to 100+ episodes/day [3], but patients and doctors frequently are not aware of them. In less than 10% of cases, LGI1 autoimmunity is linked to malignancy, such as small cell lung cancer and thymoma, with an associated dearth of data on its incidence [4].

We report a case of LGI 1 encephalitis that presented to the ED as a delayed paraneoplastic manifestation of squamous cell lung cancer in treatment-induced remission, initially diagnosed as acute delirium.

## Case presentation

A 71-year-old man visited the ED on account of subacute and worsening behavioural and cognitive changes that started couple of weeks prior to presentation. He became more forgetful and experienced visual hallucinations.

Additionally, he had multiple episodes of brief spasmodic jerks and posturing affecting the arm and face. His other reported symptom was urinary retention, which led to his presentation to the ED.

His background history was relevant for squamous cell carcinoma of the lungs, for which he received radiotherapy and four cycles of chemotherapy in 2017, and immunotherapy was completed in 2019.

During the course of the examination, he had multiple stereotypic episodes of left sided face and arm posturing, without impairment of awareness, lasting 2–4 seconds, consistent with FBDS seizures There was impaired recall with less pronounced effects on long-term memory; however, his GCS score was 15/15. No other concerning focal neurologic finding was elicited.

Complete blood count and systemic inflammatory markers were normal. He was hyponatremic, with sodium concentrations ranging from 127 to 132 mmol/l, measured serially from November 2023 to February 2024 and on treatment for immunotherapy-induced hypothyroidism and low cortisol levels. No other cause of hyponatremia was identified.

**Figure 1:**
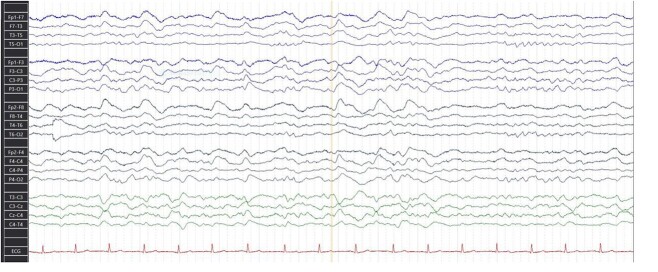
EEG of the index patient revealed generalized slow activity in keeping with encephalitis.

EEG monitoring demonstrated generalized slow waves consistent with encephalitis with no EEG correlate of FBDS (Figure 1).

**Figure 2:**
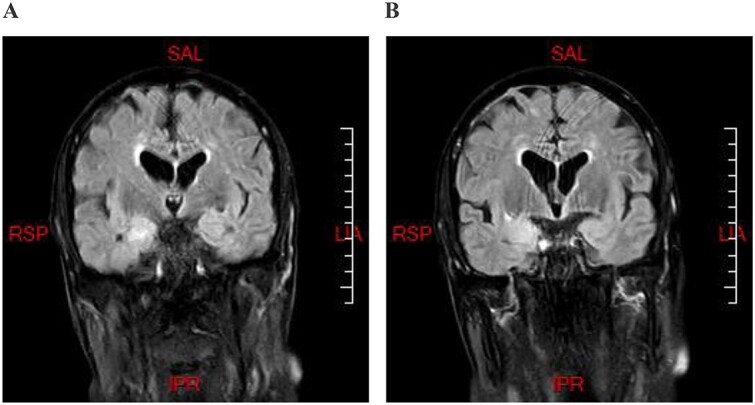
Axial brain MRI T2 FLAIR imaging demonstrating bilateral hyperintensity in the medial temporal lobe prominent on the right.

Brain MRI revealed bilateral T2 hyperintensity in the medial temporal lobe, consistent with limbic encephalitis (Figure 2). His repeat CT chest with contrast did not show any evidence of tumor activity.

**Figure 3:**
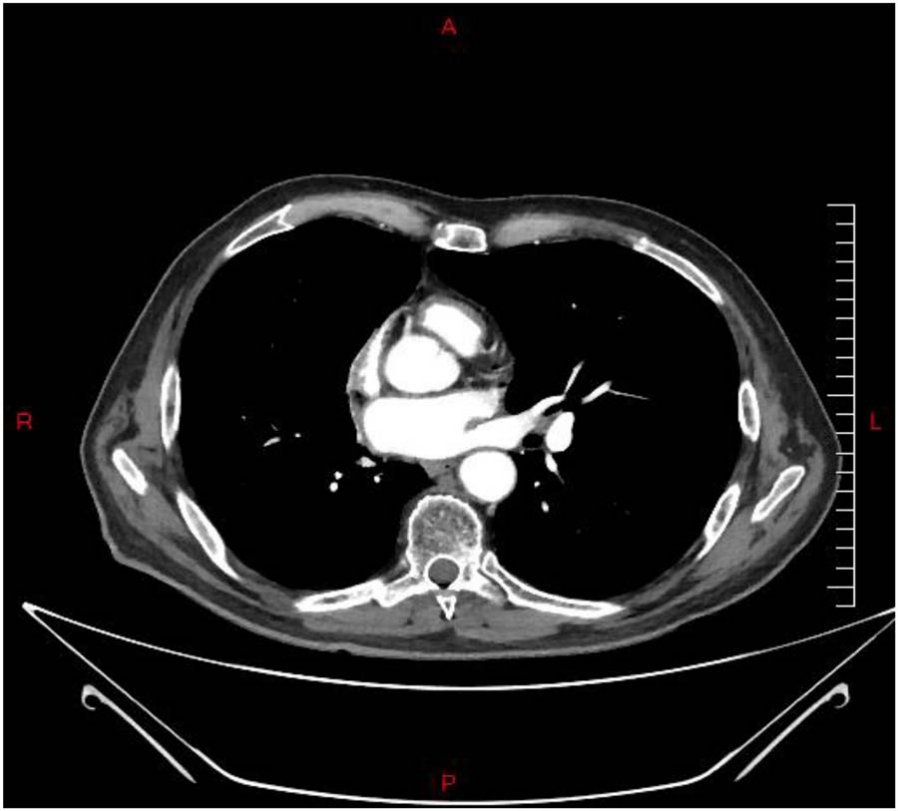
Axial contrast CT chest revealed normal finding with no evidence of tumour activity.

CSF analysis revealed mild protein elevation of 50 mg/dL with positive antibodies to the LGI1 protein, otherwise unremarkable.

He was started on an induction regime of IV methylprednisolone 1000 mg daily 5 days, then 2 weekly steroid regime of oral prednisone 1 mg/kg/d (adult, 60 mg daily maximum), 45 mg/d, 30 mg/d sequentially and a maintenance regime of 20 mg/d over the ensuing 3–6 months.

He responded to immunosuppressive therapy, as evidenced by the cessation of all FBDS on follow-up clinic review approximately a month after diagnosis.

## Discussion

A shift in perspective regarding the clinical utility of voltage-gated potassium channel (VGKC) antibodies was made possible by the discovery in 2010 of autoantibodies against the extracellular proteins LGI1 and Caspr2. There are only few case reports describing an association between squamous cell lung cancer and LGI1 autoimmune encephalitis [5]. This seems to be the first report of LGI1 encephalitis presenting following treatment-induced remission of squamous cell lung cancer, further expanding the range of paraneoplastic associations with LGI1 encephalitis and demonstrating a response to immunotherapy.

Anti-LGI1 encephalitis can be identified using the following criteria, according to the consensus of Chinese experts on the diagnosis of autoimmune encephalitis: FBDS of acute or subacute onset with progressive aggravation of limbic encephalitis symptoms; normal or mild lymphocyte pleocytosis; abnormal brain MRI signals in the unilateral or bilateral medial temporal lobe; abnormal EEG activity; and serum and/or CSF anti-LGI l antibody positivity [6]. Our case illustrated these typified symptoms. He had a relevant history of lung malignancy on remission, presented to the ED with subacute behavioural changes with an initial suspicion of acute delirium precipitated by urinary retention. Unsurprisingly, his EEG revealed an encephalopathic pattern but with no EEG correlate of FBD, which is not uncommon in LGI1 encephalitis and is thought to be due to localization of seizure focus to deeper structures such as the basal ganglia and insular or small size of the seizure onset zone, which may be difficult to detect with scalp EEG [7]. The diagnosis was confirmed by the presence of anti-LGI 1 antibody in the CSF.

LGI1 is a part of neuronal cell adhesion molecules and trans-synaptic complexes expressed in the hippocampal and temporal cortex and regulate nerve excitability and synaptic transmission [8]. AMPAR a representative target receptor for the autoantigen LGI1 is essential for the normal functioning of the limbic system of the adult brain and in regulating brain excitability and it is thought that this immune pathophysiologic processes cause a disruption in the normal homeostatic balance of neuronal excitation and inhibition, which in turn causes memory decline and seizure generation in these patients [9].

Investigational tools such as CSF studies may exhibit mild lymphocytic pleocytosis and protein elevation, however, in 50% of cases, CSF is normal. While fludeoxyglucose positron emission tomography (FDG-PET), may reveal hypo- or hypermetabolism affecting the mesial temporal structures and basal ganglia [1].

LGI 1 encephalitis is largely a non-paraneoplastic autoimmune condition, however anti-LGI1 encephalitis of paraneoplastic origin (20%), although rare, has been described especially with small cell lung Ca (SCLC) and thymoma [10]. The correlation between the typical neurological phenotype of LGI1 encephalitis and cancer, despite its rarity, and the availability of immunotherapy as a therapeutic option, implies the necessity of maintaining a low index of suspicion in these clinical contexts. Given that our patient’s cancer has remitted and his CT chest, abdomen and pelvis is negative, it is possible that the neurologic paraneoplastic autoimmunity is a delayed cross-reactive autoimmunity brought on by LGI1 antigen expression in non-neoplastic cells; however, this may also be from neoplastic cells. Thus, it is crucial to remember that in certain individuals, this could indicate residual disease or be a clue of a tumour recurrence. Therefore, active surveillance should be implemented to determine whether either of these conditions is present [6].

In the context of multiple FBDS that appear to be unrelenting and limbic encephalitis with a history of cancer, an increased likelihood that the cause is either autoimmune or paraneoplastic should be entertained, and clinicians should be cautious not to rule out LGI1 as a cause of seizures or encephalitis based solely on a negative CSF antibody result because LGI1 antibodies can be absent in spinal fluid in up to 50% of patients, even in cases that are clearly diagnosed using cell-based assays [4]. Importantly, other paraneoplastic syndrome like SCLC or non-paraneoplastic syndromes may present with limbic encephalitis with varied antibody profile. In these circumstances, the presence of specific demographic and comorbid features, such as FBDS, diarrhea, and ovarian teratoma, may initially point to the underlying disorder (LGI1 encephalitis, anti-NMDA receptor, anti-dipeptidyl-peptidase-like protein-6) [11]. Furthermore, as was the case with our patient, individuals with suspected encephalitis, especially those with cancer or in other immunocompromised state, should have an infectious screen, including a TB screen, to exclude an infectious cause for their symptoms given that their clinical phenotype, neuroradiologic, electrophysiologic, and CSF profiles may be similar [12].

Neuropsychiatric symptoms are found in 60% of cases of LGI1 encephalitis coupled with somatosensory changes and paroxysmal non-vertiginous dizziness sensation [13]. However, these findings were not elaborate on in our patient.

Research have shown that 60%–88% of patients have refractory hyponatremia which was consistent with our case with the lower level of sodium of 126.1 mmol/l Syndrome of inappropriate antidiuretic hormone secretion brought on by simultaneous LGI1 expression in the kidney and hypothalamus is most likely linked to this pathogenic mechanism [14].

In planning treatment for these individuals, especially those with seizures, aetiologies must be found early in the course of the condition because these patients with immune causes respond well to immunotherapy and typically do not respond well to antiseizure drugs. This is particularly true since early treatment with immunotherapy is linked to better results [15]. Methylprednisolone, IVIg and plasmapheresis are usually used alone or in combination as the most common first-line therapy for patient with a typical treatment regimen of Methylprednisolone 1 g daily for 5 days, IVIg 400 mg/kg/day for 5 days and plasmapheresis, with 5 exchanges over 7 to 8 days. If the response is favourable, corticosteroids and IVIg are frequently administered after the induction period on gradual tapering schedule. Other second line immunosuppressants like rituximab, azathioprine, mycophenolate mofetil may also be administered empirically when appropriate following induction [16].

## Conclusion

Testing for antineuronal antibodies depending on the clinical presentation of the patient as part of the diagnostic workup is helpful in cases of seizures with new-onset neuropsychiatric symptoms, as early diagnosis of an autoimmune syndrome in this case LG1I encephalitis can reduce years of morbidity. Thus, it is imperative that clinicians recognize immune aetiologies early by understanding and integrating the clinical and paraclinical spectrums of these presentations as undiagnosed cases, would result in missed opportunities to administer an effective therapy and consequently in poor prognosis.

### Key findings/clinical importances

Paraneoplastic syndrome in individuals with cancer in remission is rare.

Timely recognition and early initiation of treatment will likely prevent long term comorbidities such as sleep and cognitive dysfunction.

In cases of suspected paraneoplastic limbic encephalitis, sufficient surveillance and investigation should be implemented to rule out tumour recurrence and/or residual disease.

## Limitation

Whole body PET scan was not available at the time of review*.*

## CRediT statement

All authors were involved in the conceptualisation, design and revision of the manuscript.
